# Trace amounts of irinotecan found in the blood of a surgeon after performing HIPEC: what does it imply?

**DOI:** 10.1515/pp-2021-0113

**Published:** 2021-03-04

**Authors:** Wim Ceelen

**Affiliations:** Department of GI Surgery, Ghent University Hospital, Ghent, Belgium

In this issue of the journal, Saint-Lorant and coworkers report a clinical study aiming to detect the presence of irinotecan and its major metabolites (SN38 and APC) as well as total platinum (Pt) in the plasma and red blood cells of surgeons performing HIPEC [1]. Irinotecan and oxaliplatin were administered at a dose of 200 and 300 mg/m^2^, respectively, and chemoperfusion was performed at 43 °C during 30 min. Of note, the combination of oxaliplatin and irinotecan is rarely used for HIPEC nowadays, since a comparative study showed that compared with oxaliplatin alone, the combination with irinotecan resulted in higher surgical morbidity without any benefit in cancer outcome [2]. During the study, which was performed from 2015 to 2018, adequate protection measures were taken, including triple gloves, FFP category three mask, and smoke evacuation. The results showed that, out of 19 plasma samples taken at 18 h (median) after completion of HIPEC, 15 were contaminated with irinotecan (median concentration 100 pg/mL). In addition, seven samples were contaminated with platinum, but at low concentrations (<16 pg/mL).

The results highlight the environmental and potential health risks associated with the manipulation of hazardous compounds. The use of antineoplastic drugs has increased rapidly over the past decades, resulting not only in contamination of surfaces and floors in hospitals, but also in the release of these drugs in the wastewater of hospitals, and henceforth in the surface water and food chain [3]. Exposure of healthcare workers to antineoplastic agents through skin contact, ingestion, or inhalation has been associated with DNA damage and with adverse reproductive outcomes [4], [5]. According to a recent systematic review by the U.S. National Toxicology Program (available at https://doi.org/10.22427/NTP-MGRAPH-5), there is a moderate level of evidence that occupational exposure to chemotherapy is associated with an increased risk of spontaneous abortion and structural DNA damage. However, the evidence on the risk of cancer was found to be inadequate. Nevertheless, the findings highlight the importance of systematic environmental assessment and biomonitoring programs based on analytical chemistry, which are currently not performed in most hospitals.

The use of chemotherapy in the operating room during HIPEC and PIPAC has prompted several toxicological studies. These allow to conclude that exposure of health care personnel is negligible with adequate precautions [6], [7]. However, contamination of floors, shoes, keyboards, and other surfaces has been detected. In addition, patient urine and abdominal drainage fluids can be a source of contamination in the ICU and hospital wards [8]. The finding of trace amounts of Pt, a known environmental pollutant, in the study of Saint-Lorant bears little clinical significance. Indeed, Ndaw et al. found no differences in urinary Pt between a control group and the medical staff involved in HIPEC and PIPAC procedures, highlighting the fact that the general population is exposed to Pt, specifically in urban environments.

The presence of irinotecan and two of its metabolites in the surgeon’s blood is of uncertain significance. A major drawback in the interpretation is the lack of a control group. Strikingly, the authors mention in the discussion that irinotecan was found in a plasma sample from a surgical resident who did *not* participate in any HIPEC procedure, suggesting that the source of contamination of the surgeon may lie elsewhere. Of note, similar to Pt, irinotecan can be considered an environmental pollutant, since the presence of the drug has been repeatedly reported in hospital effluents and wastewater treatment plants [9]. Also, the clinical risks of trace amounts of irinotecan are doubtful—as a comparison, the mean plasma concentration after IV treatment with irinotecan is 10 mg/L, which is 100,000 times higher than the median concentration found in the surgeon’s blood.

In conclusion, the results of the work of Saint-Lorant highlight the need for systematic monitoring of the workplace environment and potential exposure of healthcare personnel using standardized, readily available, and reproducible analytical assays. However, until further data become available, they do not put into question the safety of intraoperative chemotherapy administration, provided adequate protective measures are taken.
